# Integrated analysis of transcriptome and miRNAome reveals the heat stress response of *Pinellia ternata* seedlings

**DOI:** 10.1186/s12864-024-10318-x

**Published:** 2024-04-23

**Authors:** Chen Bo, Mengmeng Liu, Qian You, Xiao Liu, Yanfang Zhu, Yongbo Duan, Dexin Wang, Tao Xue, Jianping Xue

**Affiliations:** 1 Anhui Provincial Engineering Laboratory for Efficient Utilization of Featured Resource Plants, College of Life Sciences, Huaibei Normal University, Huaibei, 235000 China; 2 Huaibei Key Laboratory of Efficient Cultivation and Utilization of Resource Plants, College of Life Sciences, Huaibei Normal University, Huaibei, 235000 China; 3 College of Agriculture and Bioengineering, Heze University, Heze, 274000 China

**Keywords:** *Pinellia ternata*, Heat stress, Transcriptome, miRNAs, Regulation

## Abstract

*Pinellia ternata* (Thunb.) Briet., a valuable herb native to China, is susceptible to the “sprout tumble” phenomenon because of high temperatures, resulting in a significant yield reduction. However, the molecular regulatory mechanisms underlying the response of *P. ternata* to heat stress are not well understood. In this study, we integrated transcriptome and miRNAome sequencing to identify heat-response genes, microRNAs (miRNAs), and key miRNA-target pairs in *P. ternata* that differed between heat-stress and room-temperature conditions. Transcriptome analysis revealed extensive reprogramming of 4,960 genes across various categories, predominantly associated with cellular and metabolic processes, responses to stimuli, biological regulation, cell parts, organelles, membranes, and catalytic and binding activities. miRNAome sequencing identified 1,597 known/conserved miRNAs that were differentially expressed between the two test conditions. According to the analysis, genes and miRNAs associated with the regulation of transcription, DNA template, transcription factor activity, and sequence-specific DNA binding pathways may play a major role in the resistance to heat stress in *P. ternata*. Integrated analysis of the transcriptome and miRNAome expression data revealed 41 high-confidence miRNA-mRNA pairs, forming 25 modules. MYB-like proteins and calcium-responsive transcription coactivators may play an integral role in heat-stress resistance in *P. ternata*. Additionally, the candidate genes and miRNAs were subjected to quantitative real-time polymerase chain reaction to validate their expression patterns. These results offer a foundation for future studies exploring the mechanisms and critical genes involved in heat-stress resistance in *P. ternata*.

**Supplementary Information:**

The online version contains supplementary material available at 10.1186/s12864-024-10318-x.

## Introduction

Global warming has led to an increased incidence of extreme heat, which is a main factor affecting plant yield [1]. Besides the impact on the plant phenotype, heat stress disrupts cellular homeostasis, severely affecting their growth and development, leading to death in some cases [2]. Heat stress, particularly high-temperature stress, inhibits a wide range of physiological and biochemical responses in plants, including changes in water use, cell membrane stability, photosynthesis, and secondary metabolite and phytohormone levels [3]. Consequently, examining plant reactions to high temperatures will enhance our understanding of thermal adaptation at the molecular level and advance the identification of heat-resistant cultivars through genetic enhancement.

Plants possess distinct adaptive mechanisms that protect them from environmental stress [4]. These mechanisms are typically governed by intricate regulatory networks comprising multiple genes. Exploring gene interactions and genetic mechanisms is challenging because traditional research methods are often limited to the structure and function of genes. Transcriptome sequencing is a crucial approach for analyzing gene expression levels, identifying differentially expressed genes, exploring functional genes, and studying genetic evolution [5]. The technology has been exploited to study plant hormone signaling pathways, transcription factors (TFs), and protein kinases that respond to heat stress in a wide range of plants [6–8].

MicroRNAs (miRNAs) are produced from a single-stranded RNA precursors with a hairpin secondary structure and typically ranges from 20 to 24 nucleotides in size [9]. Plant miRNAs have a variety of biological functions and are involved in regulating plant growth and development, hormone signaling, and environmental stress responses [10]. They act as epigenetic regulators and negatively regulate gene expression at the post-transcriptional level, either by directly cleaving the target mRNA or by inhibiting the translation of the target gene by recognizing and binding to their mRNAs [11, 12]. High-throughput sequencing allows the easy and rapid identification of miRNAs in plants, which can be further combined with transcriptome and bioinformatics analyses to screen for miRNA target genes and enrich their regulatory networks.

*Pinellia ternata* (Thunb.) Briet. is a perennial herb of the Araceae family that has been traditionally used in Chinese medicine [13]. Its tuber is highly valued for medicinal uses and known for its antiemetic, antitussive, analgesic and sedative properties [14, 15]. The complex components of *P. ternata*, including alkaloids, organic acid, polysaccharose, proteins, and nucleosides, have been well-documented [16, 17]. Among these, alkaloids are recognized as the main active ingredients and believed to exert anticancer effects [18]. Currently, the market demand for *P. ternata* is increasing. However, owing to the changing climate and increased indiscriminate logging, natural resources of *P. ternata* have been significantly reduced [19]. Consequently, artificial cultivation has become the primary production method. High temperatures are one of the most influential factors in the average growth of *P. ternata*; Atmospheric temperature above 30 °C can cause “sprout tumble” in plants, where the above-ground parts rapidly wilt and die [20]. The “sprout tumble” in *P. ternata* is a response to heat stress rather than a necessary physiological process [21]. One small heat-shock protein (sHSP), two heat-shock proteins (HSPs), and one stearoyl-ACP-protein desaturase (SAD) were found in an SSH library of *P. ternata* under heat stress [22]. The overexpression of *PtsHSP17.2* in tobacco increased the water retention and antioxidant capacity of transgenic plants, leading to a significant increase in heat tolerance [23]. In addition, overexpression of *PtSAD* in *P. ternata* reduced heat tolerance and increased the proportion of unsaturated fatty acids in transgenic plants [24]. Recent studies have shown that the PtNAC66 transcription factor enhances tolerance to heat stress in transgenic *Arabidopsis* by binding to and repressing the expression of the promoter regions of CYP707A3, MYB102, and NAC055, respectively [25]. Although progress has been made, our understanding of the molecular regulatory mechanisms underlying heat stress tolerance in *P. ternata* remains limited.

In this study, high-throughput sequencing was performed to investigate the changes in the transcriptome and miRNAs of *P. ternata* under heat stress. We screened for differentially expressed genes (DEGs) and identified miRNAs involved in the heat-stress response of *P. ternata*, providing a new theoretical basis for studying the molecular mechanisms and key resistance genes associated with “sprout tumble.”

## Materials and methods

### Plant material, growth conditions and treatments

The *P. ternata* variety used in the study was tested using tubers 1 cm in diameter from the Huaibei Normal University’s experimental field. The tubers were planted in pots and placed in a constant temperature-and-light incubator with a temperature of 25 ℃, light intensity of 4,000 lx, and a photoperiod of 16 h of light followed by 8 h of darkness. The conditions for the heat treatment of *P. ternata* have been previously described [24]. Seedlings that displayed consistent growth trends and reached a height of 15 cm were selected for the heat treatment experiment. The selected seedlings were subjected to a temperature of 40 ℃ for either 0–24 h while keeping all other conditions the same as those in the control group. Samples of leaves were collected, immediately frozen in liquid nitrogen, and stored at − 80 ℃ for further analysis. The experiments were performed three times, with three biological replicates per treatment.

### DAB, NBT staining and biochemical index determination

Leaves of *P. ternata* seedlings were soaked in 1 mg/mL DAB and 0.5 mg/mL NBT staining buffers, vacuum-infiltrated for 20 min, stained in the dark at 28 ℃ for 8 h, and boiled in ethanol: lactic acid: glycerin (3:1:1) for 5 min before observation [26]. The specimens gathered before and after heat treatment were examined, with the concentration of chlorophyll in the foliage and stalks gauged using the Lichtenthaler method [27]. Malondialdehyde (MDA) content and peroxidase (POD) activity in the plant materials were assessed according to previously published methods [23].

### Total RNA extraction, cDNA library construction and sequencing

Leaves from *P. ternata* seedlings were collected under normal and heat-treated (40 ℃ for 24 h) conditions at the three-leaf stage. TRIzol reagent (Sangon Biotech, Shanghai, China) was used to extract total RNA from the tissue samples. To ensure the accuracy of the experiment, the concentration and purity of RNA were determined using a NanoDropND-1000 spectrophotometer (Thermo Fisher Scientific, Waltham, MA, USA). The integrity of total RNA was assessed using an Agilent 2100 bioanalyzer (Agilent Technologies, Santa Clara, CA, USA). Next, 1.0 µg RNA from each group was randomly fragmented. Equal amounts of RNA from each sample were sequenced on an Illumina HiSeq2500 sequencer (Illumina, San Diego, CA, USA) at the Sangon Biotech Company (Shanghai, China) to construct strand-specific RNA-seq libraries. For the RNA-seq analysis, three biological replicates were used for each condition.

### Data processing and identification of DEGs

The initial output generated by the sequencing instrument was designated as raw reads. Raw reads obtained from each sample were subjected to a series of filtering steps. First, reads containing more than 10% of unknown nucleotides were removed. Additionally, reads with more than 40% low-quality bases were discarded. Finally, adapter sequences were eliminated from the reads using the Illumina adapter list. The remaining good-quality reads were subsequently subjected to de novo assembly using the Trinity software [28]. Transcript abundance reflects gene expression levels; the higher the transcript abundance, the higher the level of gene expression. To make the estimated gene expression levels comparable between different samples, the TPM algorithm in the Salmon software was used to measure the relative abundance of transcripts in each group of samples. DEGs in the samples were screened by DESeq; *q*-value < 0.05 and log_2_(fold change) > 1 were used as screening criteria to determine the significance of expression levels.

### Gene annotation

To annotate the identified genes, alignments were performed based on the following databases: NCBI non-redundant protein sequences (NR) (http://ncbi.nlm.nih.gov/), NCBI nucleotide sequences (NT) (http://ncbi.nlm.nih.gov/), Conserved Domain Database (CDD) (https://www.ncbi.nlm.nih.gov/cdd/), Swiss-Prot (http://www.uniprot.org/), TrEMBL (http://www.expasy.ch/sprot/), Gene Ontology (GO) (http://www.geneontology.org/), Kyoto Encyclopedia of Genes and Genomes (KEGG) (http://www.genome.jp/kegg/), euKaryotic Ortholog Groups (KOG) (http://www.ncbi.nlm.nih.gov/KOG/), and Pfam (http://pfam.xfam.org/) using BLAST version 2.2.26 [29].

### Construction of small RNA libraries and sequencing

Six samples (C: control, G: heat stress; each with three biological replicates) were harvested for small RNA library construction and sequencing. Total RNA was extracted from the samples using TRIzol reagent (Sangon Biotech, Shanghai, China). A total of 1.0 µg of RNA was utilized in the preparation of a small RNA library, following the guidelines provided by the TruSeq Small RNA Sample Prep Kits (Illumina, San Diego, USA). Single-end sequencing, with a length of approximately 50 bp, was performed using an Illumina Hiseq2500 at Sangon Biotech Company (Shanghai, China).

### Identification of miRNAs and prediction of their target genes

The raw data was processed using cutadapt software to eliminate the 3’-end connector (TGGAATTCTCGGGTGCCAAGGAACTC) and retain reads within the length range of 17–35 bp after connector removal. The reads were processed using the trimmomatic software to exclude bases with a quality score below 20 at both the 5’- and 3’-ends. Additionally, reads including four consecutive bases with an average quality value below 20 and reads shorter than 17 bases were filtered out. Raw data were filtered by removing joints and low-quality sequences to generate clean data. Clean reads were compared with the rRNA, sRNA, snRNA, and snoRNA annotation data from the RFAM database using BLASTN. The aligned reads were filtered according to the subsequent comparative criteria: gapopen = 0, evalue < 0.01, and mismatch ≤ 1. Any reads mapped to these databases were excluded from the analysis.

The miRNA levels in the heat-treated samples were analyzed for differential expression using the edgeR algorithm, with read counts serving as input data [30]. To remove miRNAs with low expression levels before performing differential analysis, the absolute value of log_2_(FoldChange) > 1 and *p*-value < 0.05 were set as screening criteria. psRNATarget (a plant small RNA target analysis server) (http://plantgrn.noble.org/psRNATarget/) (V2, 2017 release), based on the imperfect complementarity between miRNA sequences and their target genes, was used to predict miRNA target genes with default parameters and a maximum expectation value of four [31].

### Quantitative RT-PCR analysis

To validate the transcriptome data, the SYBR green-based qPCR method was employed, and the *Pt18SrRNA* gene was used as an internal control for normalization [24, 32]. Primer sequences used for qRT-PCR are listed in Supplemental Table S1. miRNA expression was assessed using the Mir-XTM miRNA First-Strand Synthesis Kit (Clontech) for reverse transcription. Primers were specifically constructed for the six selected miRNAs, with the U6 snRNA serving as an internal control (Supplemental Table S1). qRT-PCR analyses were conducted three times, each containing three replicates for all genes. The 2^−ΔΔCT^ method was used to calculate the relative expression level of each gene [33].

## Results

### Phenotypic analysis under heat stress

**Fig. 1:**
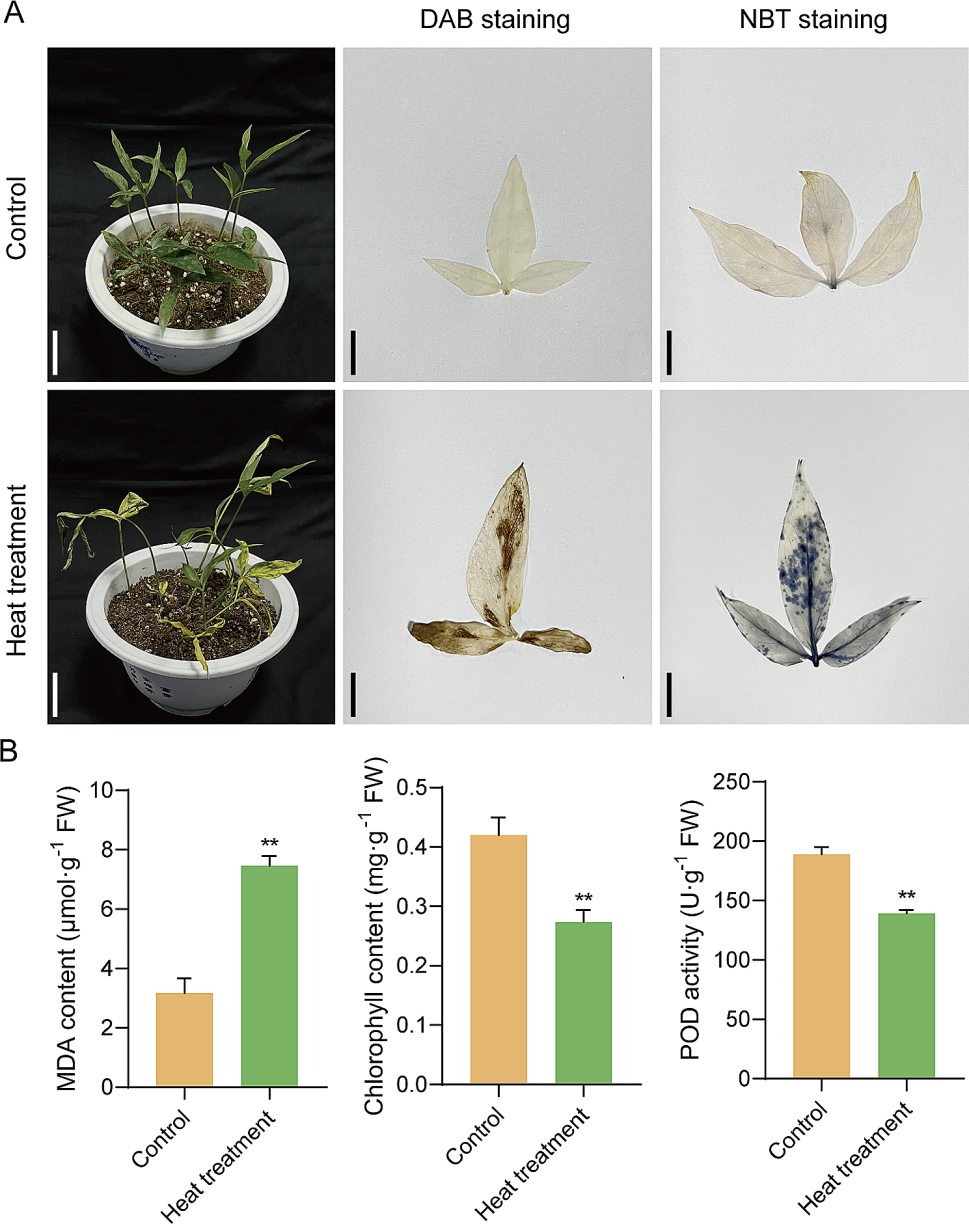
Physiological responses of *Pinellia ternata* seedlings before and after heat treatment. (**A**) *P. ternata* seedlings phenotypes in response to heat stress. Plants were grown in soil to the three-leaf stage and subjected to a 40 ℃ stress treatment for 24 h (left, scale bar = 4 cm). DAB and NBT staining from the same part of *P. ternata* seedlings before and after heat treatment (right, scale bar = 15 mm). (**B**) MDA and chlorophyll contents, and POD activities in *P. ternata* seedlings under control and heat treatment for 24 h. Except where noted, all data are presented as mean (*n* = 3) and standard deviation. Data were analyzed by Student’s *t*-test and one-way _ANOVA_. * and ** indicate significant differences between control and heat treatment plants at the *P* < 0.05 and *P* < 0.01 levels, respectively

Wild-type *P. ternata* seedlings of uniform size at the three-leaf stage were subjected to stress treatment at 40 ℃ for 24 h and evaluated for their phenotypic response to heat stress. As shown in Fig. 1A, P. *ternata* seedlings subjected to heat stress showed no significant changes compared to control conditions. However, after 24 h of heat treatment, plants exhibited obvious deleterious phenotypes, such as curled and wilted leaves, shortened plant height, and growth inhibition, compared to the control plants. Reactive oxygen species (ROS) are produced in plants in response to stresses, such as heat stress. To further understand the cellular damage induced by ROS accumulation, MDA content, chlorophyll content, and POD activities in *P. ternata* seedlings were compared before and after heat treatment. The MDA content of the heat-treated *P. ternata* seedlings was two-fold higher than that of the control seedlings. Conversely, chlorophyll content and POD activity of the heat-treated plants were significantly lower than those of the control plants (Fig. 1B). This indicated that wild-type *P. ternata* seedlings exhibited a high degree of susceptibility to heat stress.

### Transcriptome sequencing in *P. ternata* under heat stress

To examine the gene expression patterns of *P. ternata* under heat stress, six libraries were constructed from two leaf samples, each with three biological replicates (C: control; G: heat stress). Following the removal of low-quality reads, each library’s total reads and total bases ranged from 42.34 million to 53.72 million and 6.10 billion to 7.69 billion, respectively. The Q30 and GC contents were consistently high across all libraries, with values ranging from 93.83 to 94.36% and 55.64–58.43%, respectively, indicating the exceptional quality of the transcriptome sequencing data (Supplemental Table S2). A comprehensive collection of 574,168 transcripts was obtained from cDNA libraries. These transcripts were subsequently filtered and assembled, resulting in the formation of 208,217 unigene clusters. Notably, these clusters exhibited an N50 value of 552 bp. A comprehensive summary of the transcriptome sequencing analysis of *P. ternata* is presented in Table 1.

**Table 1:** Summary of *P. ternata* transcriptome sequencing

Index	Transcript	Unigene
All	574168	208217
N50 (bp)	523	552
N90 (bp)	245	243
Max Length (bp)	13532	13532
Min Length (bp)	201	201
Total Length (bp)	269678720	99677674
Average Length (bp)	469.69	478.72

All assembled unigene clusters were aligned against the GO, KEGG, Pfam, SwissProt, TrEMBL, CDD, KOG, NR, and NT databases using DIAMOND 23 with a threshold E-value of < 0.00001 [28]. The statistical results of the six authoritative databases are listed in Table 2.

**Table 2:** Statistical results from the DIAMOND 23 annotation.

Database	Number of genes	Percentage(%)
Annotated in GO	52926	25.42
Annotated in KEGG	5487	2.64
Annotated in PFAM	20945	10.06
Annotated in Swissprot	43907	21.09
Annotated in TrEMBL	60671	29.14
Annotated in CDD	32818	15.76
Annotated in KOG	20812	10
Annotated in NR	62175	29.86
Annotated in NT	24688	11.86
Annotated in at least one database	68151	32.73
Annotated in all database	2306	1.11
Total genes	208217	100

### Analysis of DEGs

**Fig. 2:**
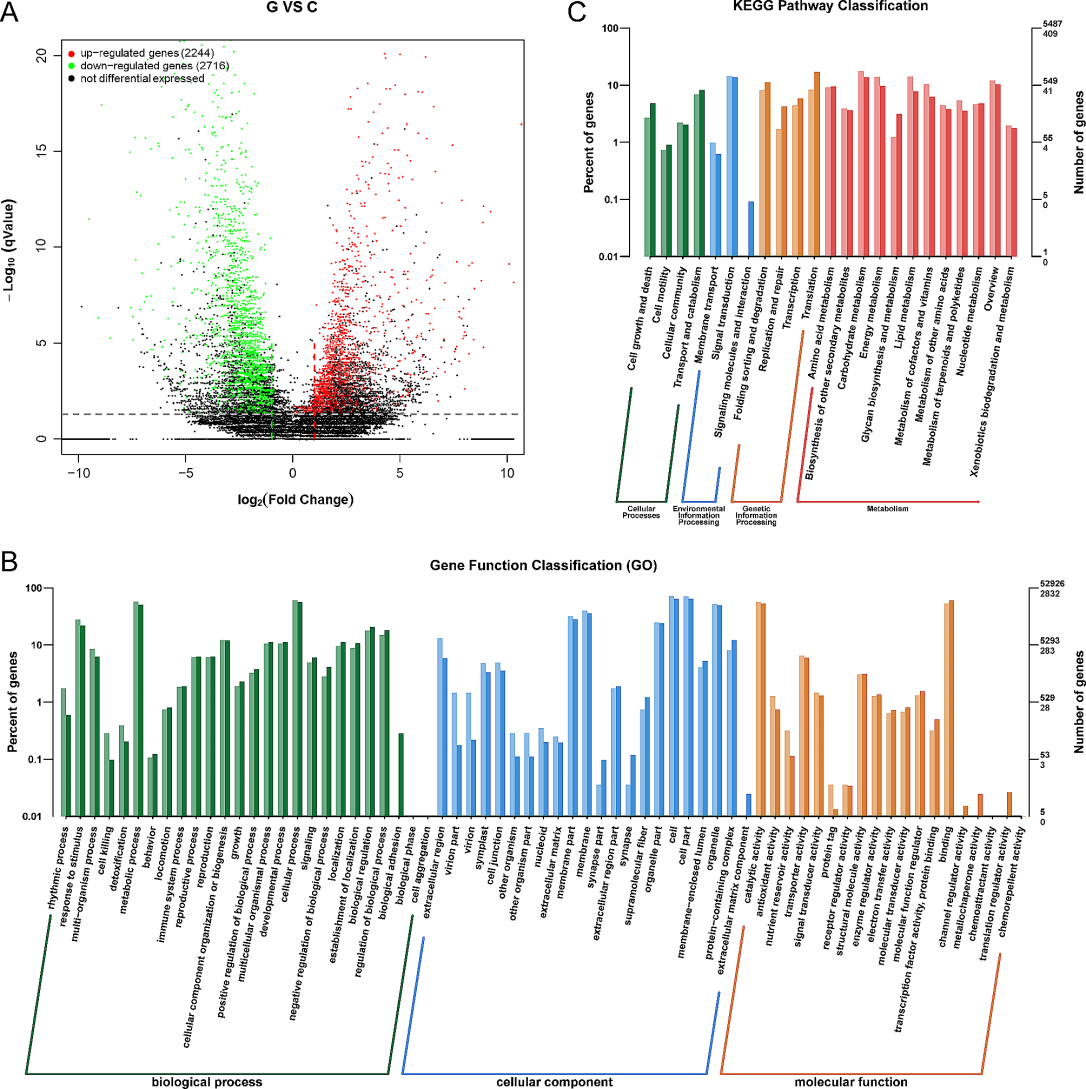
Transcriptome sequencing analysis of G and C. (**A**) scatter plot of DEGs in G vs. C. Significantly differentially expressed genes are represented by red dots (up-regulated) and green dots (down-regulated), whereas non-differentially expressed genes are represented by black dots. (**B**) Gene ontology (GO) classification of the DEGs in *P. ternata* seedlings under heat stress. The ordinate is the enriched GO term, and the abscissa is the percent and number of differentially expressed genes in this term. Different colors are used to distinguish biological processes, cellular components, and molecular functions. (**C**) Kyoto Encyclopedia of Genes and Genome (KEGG) enrichment of the DEGs in *P. ternata* under heat stress. The ordinate is the enriched KEGG term, and the abscissa is the percent and number of differentially expressed genes in this term. Different colors are used to distinguish biological processes, cellular components, and molecular functions

Three comparisons of the two test conditions (C and G) were conducted to identify genes differentially regulated by heat stress in *P. ternata*. The cluster heat map (Supplemental Fig. S1A) and PCA plots (Supplemental Fig. S1B) demonstrated a high degree of reproducibility of the gene expression data across the three biological replicates of each sample. Notably, transcriptome expression levels exhibited contrasting patterns before and after subjecting *P. ternata* seedlings to heat treatment. As is evident from the scatter plot, the number of significantly upregulated transcripts (2,244) was lower than that of downregulated transcripts (2,716) among the 4,960 DEGs (Fig. 2A; Supplemental Table S3).

### Functional annotation and enrichment analysis of the DEGs

GO enrichment analysis was performed to determine the functions of the identified DEGs, and 7,028 DEGs were annotated to 4,610 GO terms. Notably, we observed significant enrichment (*p* < 0.05) of 817 GO categories in *P. ternata* under heat stress (Supplemental Table S4). As depicted in Fig. 2B, the significantly enriched biological processes (BPs) encompassed cellular processes (GO:0009987), metabolic processes (GO:0008152), responses to stimuli (GO:0050896), and biological regulation (GO:0065007); the most significantly enriched cellular components (CCs) were cells (GO: 0005623), cell parts (GO:0044464), organelles (GO:0043226), and membranes (GO:0016020). In addition, catalytic activity (GO:0003824) and binding activity (GO:0005488) were significantly enriched in the molecular function (MF) categories.

To conduct a more comprehensive examination of the biological functions of the DEGs, pathway-based analysis was performed using KEGG pathway enrichment analysis. Carbohydrate metabolism, signal transduction, lipid metabolism, and energy metabolism were the most enriched pathways in *P. ternata* under heat stress (Fig. 2C). A total of 409 DEGs were assigned to 158 distinct pathways. Among these pathways, 78 were significantly enriched (*p* < 0.05) (Supplemental Table S4).

### Several genes encoding TFs were differentially expressed

**Fig. 3:**
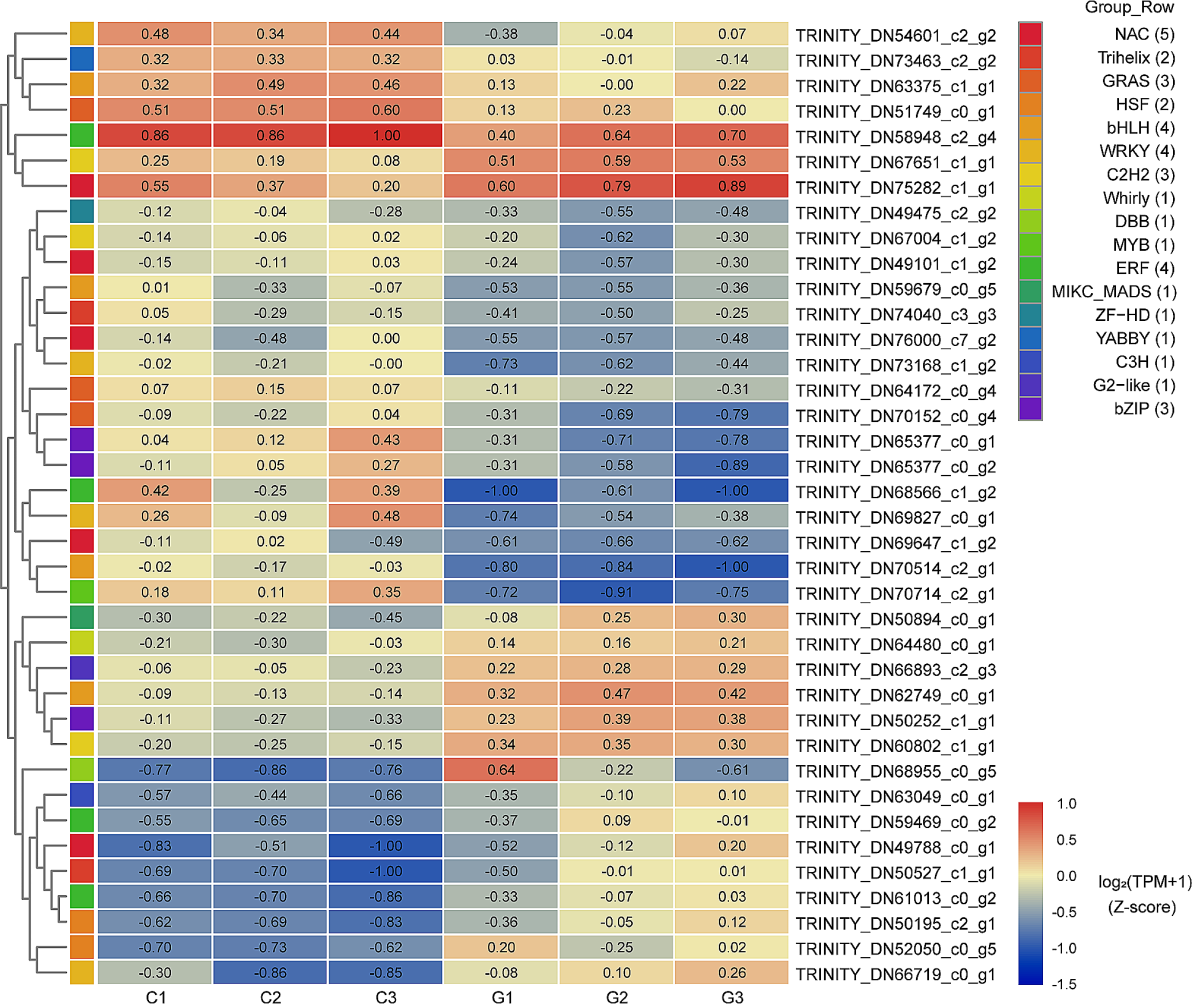
Heatmap of the regulatory multiples of 38 transcription factors with different regulatory trends in the comparisons of control and heat stress treatments. Transcription factors of the same family are indicated by squares of the same color, and the number of genes is attached to the gene family name. The colored bars represent the 38 transcription factor values of control and heat treatment [log_2_(TPM + 1)]

TF activity and protein binding (GO:0000988) were also important GO terms (Fig. 2B). In this study, 4,960 genes were identified, of which 38 encoded TFs belonging to 17 families. The TF families with the highest representation were No apical meristem, *Arabidopsis* transcription activation factor1/2, and Cup-shaped cotyledon2 (NAC), with a count of five. This was followed by basic helix-loop-helix (bHLH), APETALA2/Ethylene responsive factor, and WRKY TF, each with a count of four (Fig. 3). The vast majority of TFs showed opposite expression trends under control and heat treatment conditions, suggesting that the response to heat stress in *P. ternata* seedlings is regulated by a combination of factors.

### Validation of RNA-Seq analysis by quantitative real-time PCR (qRT-PCR)

To validate the reliability of the heat stress-related gene expression data obtained from the RNA-Seq analysis of *P. ternata* seedlings, eight DEGs with different roles in the heat-stress response were selected from the control and heat-treated samples for qRT-PCR analysis (Supplemental Fig. S2 and Table S1). The trends in qRT-PCR and transcriptome sequencing results were consistent, further demonstrating the reliability of the transcriptome sequencing data.

### MicroRNAome for heat stress responses to ***P. ternata***

**Fig. 4:**
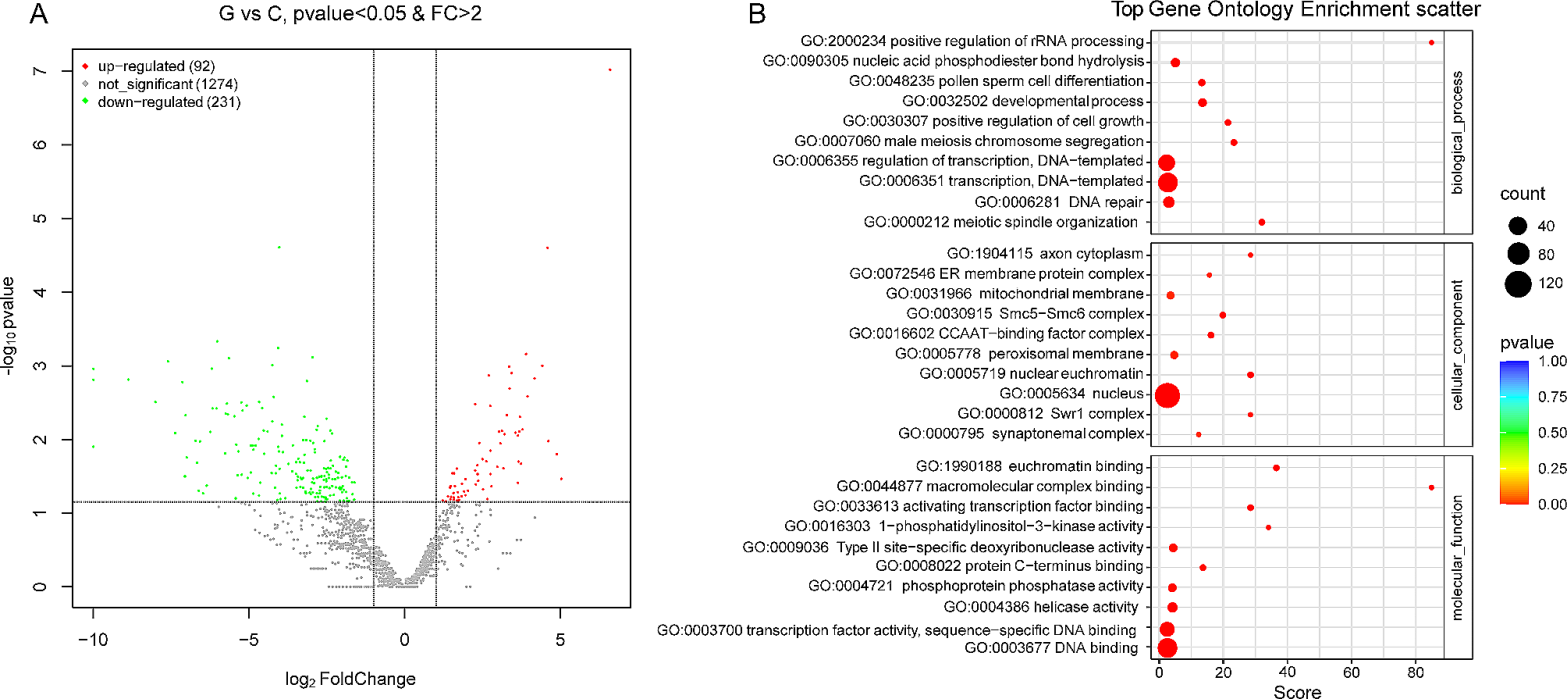
Analysis of differentially expressed miRNAs (DEMs) between G and C. (**A**) The number of DEMs in response to heat stress treatments in *P. ternata* seedlings. (**B**) Gene ontology (GO) enrichment analysis of stress-responsive DEMs under heat treatments. The ordinate is the enriched GO term, and the abscissa is the percent and number of differentially expressed genes in this term. Different colors are used to distinguish biological processes, cellular components, and molecular functions

To comprehensively evaluate the influence of heat stress on the transcript profiles, six small RNA libraries were constructed. Following the removal of low-quality reads, each library’s total reads and total bases ranged from 1.50 million to 3.11 million and 0.36 billion to 0.72 billion, respectively. The Q30 and GC contents were consistently high across all libraries, with values ranging from 96.96 to 97.09% and 45.09–51.48%, respectively, indicating the exceptional quality of the miRNA sequencing data (Supplemental Table S5). PCA was conducted on three biological replicates of each sample, and the results demonstrated a strong connection (Supplemental Fig. S3), demonstrating high dependability of the replicates. The sequences of the clean reads obtained from the experiment were compared with those of miRNAs in plants such as *Arabidopsis thaliana* [34], *Oryza sativa* [35], and *Medicago truncatula* [36], sourced from from miRbase. A total of 1,597 known/conserved miRNAs were identified across all samples (Supplemental Table S6). Unaligned readings were analyzed to identify new miRNAs. Following the exclusion of small RNAs that did not satisfy the established criteria for plant miRNAs, finally a total of 92 upregulated and 231 downregulated differentially expressed miRNAs (DEMs) was obtained (Fig. 4A; Supplemental Table S7).

### Identification of the potential target genes

To gain a thorough understanding of the biological relevance of miRNAs and other short RNAs, the individual genes that they target must be identified. This information can be used to unravel the intricate regulatory network of miRNA-target interactions and their potential impact on various biological processes. We predicted 3,017 targets for 289 miRNAs that were differentially expressed during heat stress in *P. ternata* seedlings (Supplemental Table S8). Along with aly-miR4242 and bdi-miR7713-5p, 29 other miRNAs were predicted to interact with only one target gene. The remaining miRNAs exhibited a propensity to target several genes. The maximum number of targets was 117 for ppt-miR414, followed by 88 for osa-miR2102 and 87 for ppe-miR8123-3p. GO enrichment analysis was conducted to gain a deeper understanding of the potential biological functions associated with the target genes involved in heat-stress response (Fig. 4B; Supplemental Table S9). Among the enriched BPs, the most enriched GO terms were transcription, DNA-templated (GO:0006351) and regulation of transcription, DNA-templated (GO:0006355). In the cellular component group, the nucleus (GO:0005634) was the most overrepresented term. Among the enriched MFs, the most significant GO terms were DNA binding (GO:0003677) and transcription factor activity, sequence-specific DNA binding (GO:0003700). These observations suggest that the identified miRNAs may have a significant impact on DNA binding and transcriptional control within the nucleus.

### Integrated analysis of miRNAome and transcriptome

As miRNAs negatively regulate the expression of their target mRNAs through target cleavage, their expression patterns are generally inversely correlated with those of their target. We collected miRNA-target gene pairs with negative regulatory interactions from the differential miRNA-differential target gene association analysis table and combined them with the transcriptome and miRNAome data (Table 3). For example, ata-miR2275a-5p was upregulated after heat stress, whereas its potential target gene (TRINITY_DN70714_c2_g1), an MYB family gene, was downregulated under heat stress conditions. Another potential target gene (TRINITY_DN70355_c4_g2) associated with calcium response was upregulated by heat stress, and its counterpart *gma-miR172h-5p* was downregulated. Moreover, our findings indicated that heat stress led to a decrease in the expression of *ptc-miR156k*, *osa-miR166b-5p*, and *bra-miR172c-5p*, whereas *ghr-miR160* was upregulated following heat treatment. Further analysis indicated that 25 miRNAs were differentially expressed, corresponding to 41 distinct target genes, suggesting that a single miRNA has the potential to regulate multiple target genes.

**Table 3:** The miRNA-target gene pairs showed reverse expression patterns in the comparison group.

miRNA	log2FoldChange	Regulated	Target	log2FoldChange	Regulated	Annotation
ata-miR2275a-5p	2.333785162	up	TRINITY_DN70714_c2_g1	-1.825905376	down	Transcription factor, Myb superfamily
ath-miR865-5p	-3.179420997	down	TRINITY_DN69793_c0_g3	2.060074385	up	Phosphoribosylaminoimidazole carboxylase (Vigna aconitifolia)
bra-miR172c-5p	-1.872878627	down	TRINITY_DN63657_c1_g1	1.173622602	up	Paf1/RNA polymerase II complex, RTF1 component (involved in regulation of TATA box-binding protein)
cme-miR169q	-1.870743344	down	TRINITY_DN60798_c0_g1	1.303453374	up	Ornithine carbamoyltransferase, chloroplastic (Musa acuminata)
ghr-miR160	3.623852331	up	TRINITY_DN71553_c1_g1	-1.075618235	down	Uncharacterized protein LOC105044151 (Elaeis guineensis)
gma-miR172h-5p	-2.120101667	down	TRINITY_DN70355_c4_g2	3.103030198	up	Calcium-responsive transcription coactivator
gma-miR172h-5p	-2.120101667	down	TRINITY_DN48595_c0_g1	4.614621875	up	GRF1-interacting factor 1 (Phoenix dactylifera)
gma-miR396e	-4.903063906	down	TRINITY_DN74739_c4_g1	1.446181101	up	Uncharacterized protein LOC104613301 (Nelumbo nucifera)
gma-miR396h	-4.52553491	down	TRINITY_DN74739_c4_g1	1.446181101	up	Uncharacterized protein LOC104613301 (Nelumbo nucifera)
hvu-miR6214	1.596413994	up	TRINITY_DN56096_c0_g1	-2.459524584	down	Cytochrome P450 CYP4/CYP19/CYP26 subfamilies
hvu-miR6214	1.596413994	up	TRINITY_DN63516_c1_g3	-2.791599064	down	Hypothetical protein SELMODRAFT_117724 (Selaginella moellendorffii)
hvu-miR6214	1.596413994	up	TRINITY_DN71342_c3_g1	-1.913308793	down	Mavicyanin (Musa acuminata)
hvu-miR6214	1.596413994	up	TRINITY_DN65549_c0_g2	-1.648548563	down	Mavicyanin (Musa acuminata)
hvu-miR6214	1.596413994	up	TRINITY_DN66656_c2_g2	-2.662091299	down	Probable xyloglucan glycosyltransferase 9 (Musa acuminata)
hvu-miR6214	1.596413994	up	TRINITY_DN39053_c0_g1	-3.679784907	down	Proline-rich receptor-like protein kinase PERK13 (Musa acuminata)
hvu-miR6214	1.596413994	up	TRINITY_DN66595_c1_g1	-1.344644483	down	Subtilisin-like protease SBT1.7 (Capsicum annuum)
hvu-miR6214	1.596413994	up	TRINITY_DN65942_c0_g2	-1.329286242	down	Transmembrane protein 45A (Brachypodium distachyon)
hvu-miR6214	1.596413994	up	TRINITY_DN62698_c0_g1	-2.517059999	down	Uncharacterized serine-rich protein C215.13 (Nelumbo nucifera)
hvu-miR6214	1.596413994	up	TRINITY_DN72360_c2_g1	-1.126451034	down	Unnamed protein product [Coffea canephora]
mes-miR477a	-7.063544847	down	TRINITY_DN65677_c0_g3	1.67209331	up	Uncharacterized protein LOC18435300 (Amborella trichopoda)
mes-miR477a	-7.063544847	down	TRINITY_DN65677_c0_g3	1.67209331	up	Uncharacterized protein LOC18435300 [Amborella trichopoda]
mtr-miR2592ap	4.575249424	up	TRINITY_DN69416_c1_g2	-2.46793172	down	Serine carboxypeptidase-like 33 (Phoenix dactylifera)
mtr-miR319a-5p	2.25089675	up	TRINITY_DN59178_c0_g1	-1.23086805	down	1-deoxy-D-xylulose 5-phosphate reductoisomerase LOC101494630 (Cicer arietinum)
mtr-miR5561-3p	-1.81775499	down	TRINITY_DN70248_c0_g1	1.259637401	up	Uncharacterized protein LOC105056005 (Elaeis guineensis)
osa-miR166b-5p	-1.936901594	down	TRINITY_DN73459_c1_g1	2.619379805	up	39S ribosomal protein L47 (Morus notabilis)
osa-miR2931	-2.109704093	down	TRINITY_DN74783_c2_g2	1.733618537	up	Glucose-6-phosphate 1-dehydrogenase 4 (Elaeis guineensis)
osa-miR5795	1.218461156	up	TRINITY_DN61930_c1_g1	-1.302771326	down	Soflavone reductase homolog [Nicotiana sylvestris]
ppe-miR482a-5p	1.554364735	up	TRINITY_DN62981_c1_g1	-1.117358942	down	50S ribosomal protein L35, chloroplastic (Elaeis guineensis)
ppe-miR6262	-2.563930335	down	TRINITY_DN55866_c0_g1	1.905992499	up	Protein NUCLEAR FUSION DEFECTIVE 6, chloroplastic/mitochondrial (Brachypodium distachyon)
ppe-miR6275	2.409852564	up	TRINITY_DN41916_c0_g1	-2.333725712	down	Clustered mitochondria protein (Musa acuminata)
ppe-miR8123-3p	5.013405557	up	TRINITY_DN54325_c0_g2	-2.902112441	down	Expansin-A4-like (Elaeis guineensis)
ppt-miR1048-3p	-1.635244915	down	TRINITY_DN71304_c1_g1	1.634822565	up	Ankyrin-1 isoform X2 (Elaeis guineensis)
ppt-miR2083-5p	1.411864472	up	TRINITY_DN63923_c0_g3	-1.187434507	down	50S ribosomal protein L29, chloroplastic-like (Vitis vinifera)
ppt-miR2083-5p	1.411864472	up	TRINITY_DN62493_c1_g2	-1.529507641	down	Ferric reduction oxidase 7, chloroplastic-like (Elaeis guineensis)
ppt-miR414	3.687774991	up	TRINITY_DN71797_c2_g2	-1.531825334	down	Lysine histidine transporter 2 (Arabidopsis thaliana)
ppt-miR414	3.687774991	up	TRINITY_DN74214_c3_g1	-1.662038819	down	Probable strigolactone esterase D14 (Phoenix dactylifera)
ppt-miR414	3.687774991	up	TRINITY_DN54254_c1_g1	-2.022678378	down	Uncharacterized protein (Oryza punctata)
ppt-miR414	3.687774991	up	TRINITY_DN73823_c3_g4	-1.775870081	down	Uncharacterized protein LOC101498079 (Cicer arietinum)
ptc-miR156k	-3.499915672	down	TRINITY_DN75067_c4_g1	1.435481162	up	Presequence protease 1, chloroplastic/mitochondrial-like (Musa acuminata)
ptc-miR396e-3p	-6.179188792	down	TRINITY_DN68263_c0_g2	1.41738653	up	Putative lysine-specific demethylase JMJD5 (Eucalyptus grandis)
smo-miR1091	-1.943586526	down	TRINITY_DN67006_c0_g2	2.373833497	up	Uncharacterized protein LOC105032422 (Elaeis guineensis)

**Fig. 5:**
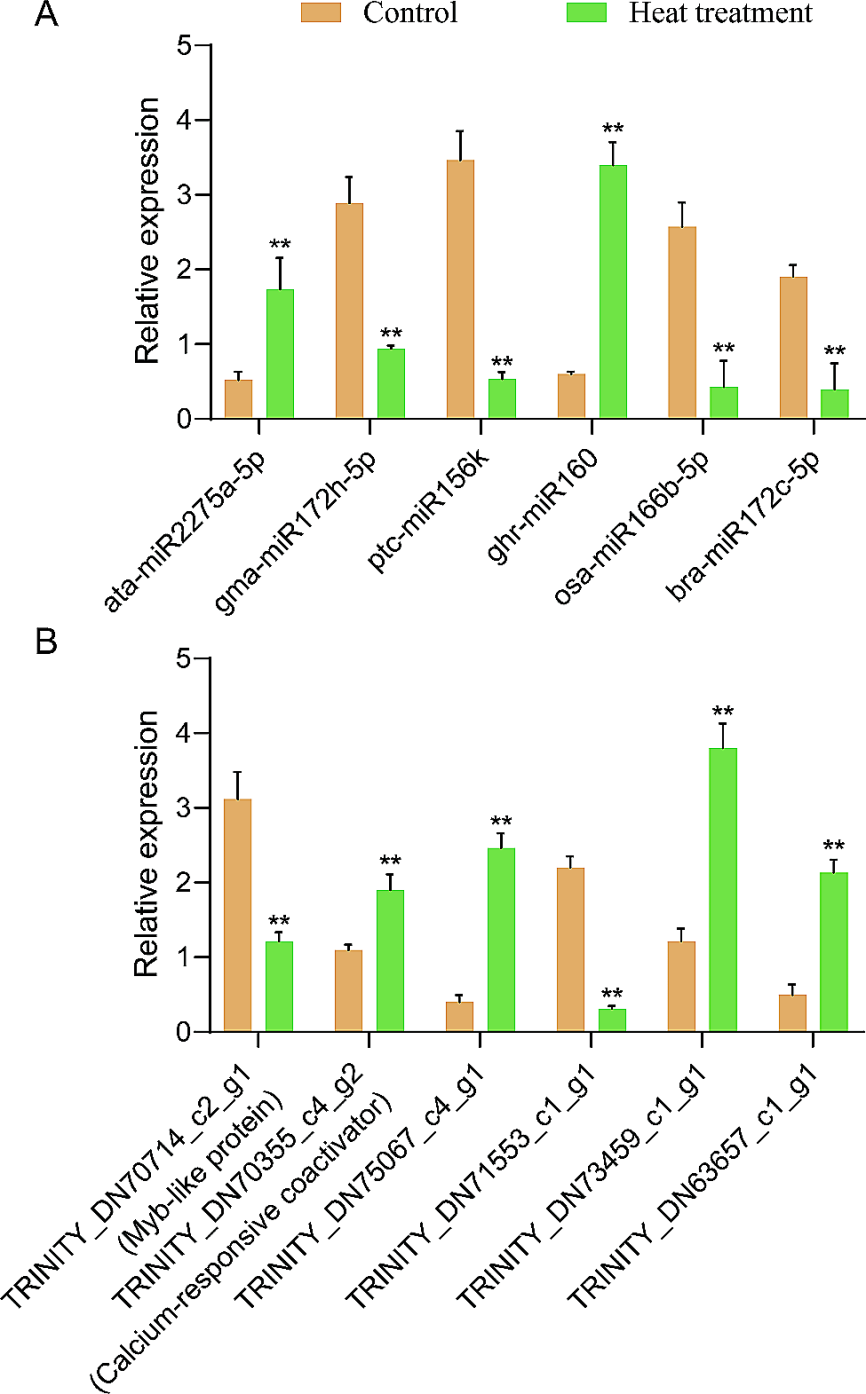
Validation of expression profile of miRNAs and their predicted target genes in *P. ternata* seedlings. Expression analysis of selected miRNAs (**A**) and one of their predicted targets genes (**B**) using qRT-PCR. Three biological replicates and two technical replicates were included in the study. Asterisks indicate significant differences between the control and the heat treatment sample (Student’s *t*-test, *P < 0.05, **P < 0.01)

**Fig. 6:**
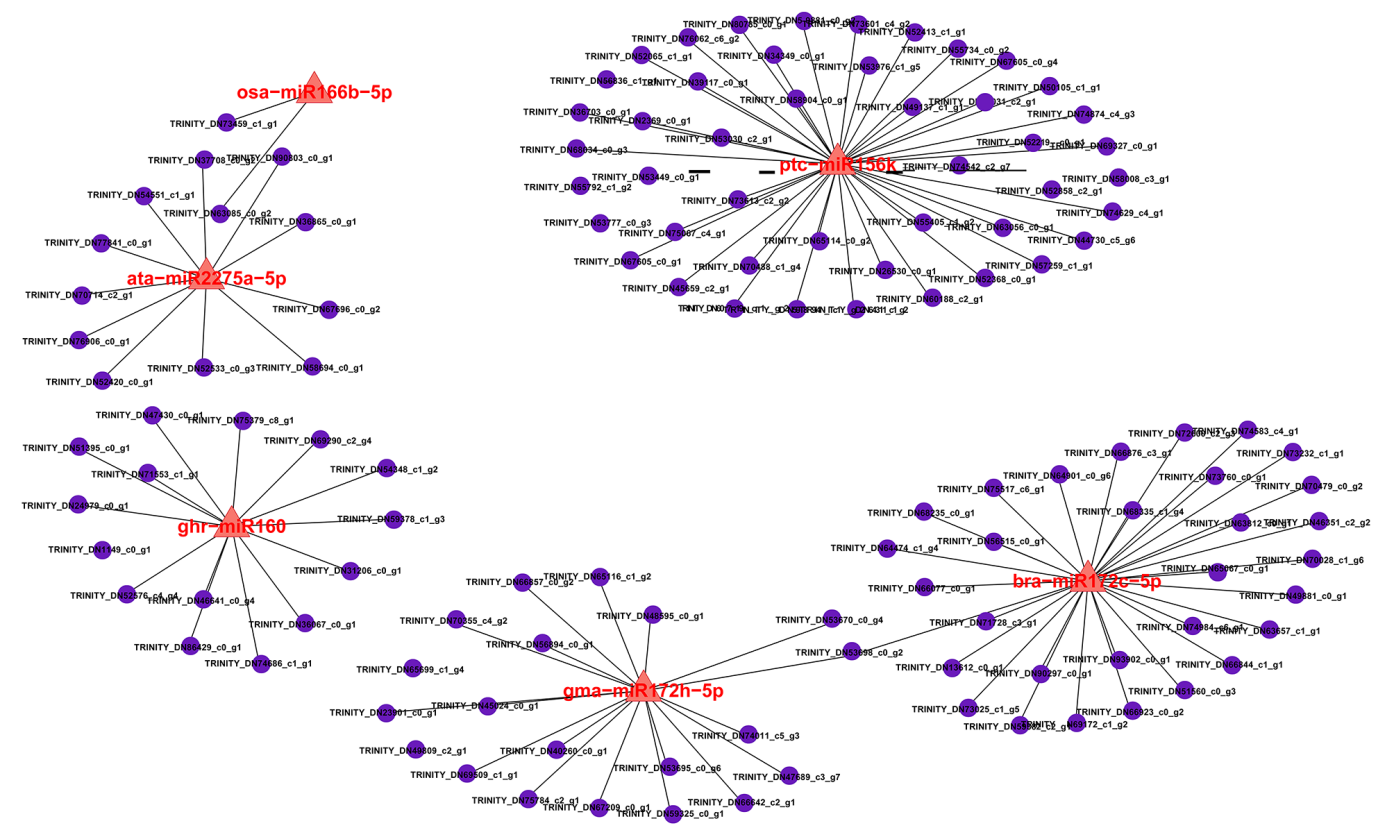
Molecular regulatory network diagram. The black lines represent the interaction between hub miRNA with their corresponding targeted genes

We performed qRT-PCR on the six aforementioned miRNAs and their corresponding possible target genes to confirm the expected regulatory link between heat stress-associated miRNAs and their targets. The qRT-PCR results agreed with those obtained from Illumina sequencing. This concurrence provides additional support for the observed inverse expression patterns of the six miRNA-target pairs investigated in our study (Fig. 5). Expression profiling analyses of miRNA-mRNA interactions demonstrated a substantial level of confidence in the prediction of target genes. To further understand the regulatory mechanisms of the miRNAs, a molecular regulatory network diagram was constructed for these six miRNAs (Fig. 6). The results showed that all the miRNAs interacted closely with the genes. However, further testing of similar pairs is required to gain insight into the response of *P. ternata* seedlings to heat stress.

## Discussion

High temperatures, whether temporary or prolonged, can induce modifications in the morphological, physiological, and biochemical properties of plants, thereby affecting plant growth and development. This can lead to a significant decrease in yield, particularly in plant species vulnerable to high temperatures [24, 37]. *P. ternata* is a heat-sensitive plant, and heat is an important limiting factor in its production. The molecular mechanisms of the response to high temperatures in model plants have been well studied; however, the mechanisms of heat-stress response have been challenging to investigate in *P. ternata* because of its susceptibility to the “sprout tumble” phenomenon caused by high temperatures. However, the absence of a genome sequence for this species poses a challenge in unraveling the underlying molecular mechanisms. Omics-based approaches, including suppression subtractive hybridization [22], proteomics [38], and transcriptomics [39, 40], has been applied to *P. ternata* for the data mining of heat-responsive factors. However, none of the genes involved in heat-stress response have been functionally verified in *P. ternata*. Therefore, it is necessary to design an integrated multi-omics approach to elucidate the molecular regulatory network of *P. ternata* in response to heat stress. Our study combined transcriptome and miRNAome data to identify miRNAs and their target genes associated with heat stress.

The transcriptome sequencing results of *P. ternata* seedlings provided valuable insights into the molecular response to heat stress. The identification of a large number of transcripts (574,168) and their assembly into unigenes (208,217) highlighted the complexity of the transcriptome and the diversity of expressed genes in this species. The identification of 4,960 DEGs indicates substantial transcriptional changes in *P. ternata* seedlings under heat stress conditions. Interestingly, the number of downregulated genes (2,716) was higher than that of upregulated genes (2,244), suggesting that the plant response to heat stress may involve the repression of certain genes or pathways, as a probable strategy to conserve energy or redirect resources toward stress response mechanisms [41]. Functional annotation of the DEGs using GO and KEGG analyses provided insights into the biological processes and pathways potentially involved in plant responses to heat stress. The involvement of DEGs in the response to stimuli and signal transduction pathways suggests that these genes play crucial roles in sensing and responding to heat stress signals. The enrichment of DEGs in plant hormone signal transduction and the MAPK signaling pathway was particularly important because these pathways play key roles in plant stress responses [42]. Because of the complexity of this regulatory mechanism, their function in plant responses to heat stress is worth-exploring.

TFs play important roles in plant responses to heat stress. Members of the TF family such as NAC [43], bHLH [44], WRKY [45], MYB [46], AP2/ERF [47] and bZIP [48] are associated with heat stress. Our analysis revealed that the downregulated members (*n* = 14) of the TF families NAC, bHLH, WRKY, and bZIP surpassed the upregulated members (*n* = 7), supporting our earlier inference. NAC TFs have been confirmed to play an important role in the response to heat stress. In rice, SNAC3 regulates the heat-stress response by adjusting the redox homeostasis by controlling the expression of ROS-associated enzyme genes [43]. We also found that the NAC gene family had the largest representation among the TF genes screened. Of the five NAC genes, two were upregulated, while three were downregulated. Further comprehensive investigations are required to ascertain whether these genes are involved in distinct stress-signaling pathways. Heat-shock transcription factors (HSFs) play a pivotal role in the adaptation of plants to heat and other stress stimuli [49]. We identified two HSF genes that were significantly altered after heat treatment of *P. ternata* seedlings. Interestingly, the expression of these two genes was reversed, suggesting the presence of a multifaceted regulatory system in *P. ternata* in response to heat stress.

Small RNAs play an important role in the response to different stresses and regulation of gene expression during plant growth and development in plants [50]. Generally, a single miRNA can target several genes, and a single gene can be regulated by several miRNAs [51]. Through miRNAome analysis, 3,017 targets from 289 miRNA families were identified from miRNA libraries. Some miRNAs that have been previously known to respond to heat stress, such as *miR156, miR160, miR166*, and *miR172*, were also detected in our study. In *Arabidopsis*, plants overexpressing *miR160* and *arf* deletion mutants have a significantly higher tolerance to high temperatures than wild-type plants [52]. In the present study, *miR160* was upregulated after heat treatment, suggesting that it might have a function similar to that in other species. *miR160, miR156*, and *miR172* also regulate plant tolerance to high temperatures by regulating the expression levels of target genes [53–55]. However, in the present study, these miRNAs were down-regulated during heat stress. One possible explanation for this phenomenon is the innate heat sensitivity of wild-type *P. ternata*, which may render it less capable of effectively enduring heat stress.

The integration of transcriptome and miRNAome expression datasets in response to heat stress in *P. ternata* led to the identification of crucial miRNA-mRNA modules. This approach provided a comprehensive understanding of the multiple regulatory networks involved in this biological process. To construct our regulatory network, we utilized only those miRNA-target pairs that showed inverse expression patterns in both our miRNAome and transcriptome datasets with high confidence. The analysis revealed that 25 miRNAs were differentially expressed, corresponding to 41 distinct target genes (Table 3). Among these, we identified an MYB-like and calcium-responsive gene that was significantly regulated by miRNAs. In *Phaseolus vulgaris*, the MYB family gene *PvPHR1* is regulated by PvmiR399, and they play an important role in plant phosphorus deficiency signaling [56]. To date, no studies have provided evidence for the involvement of miRNAs in the regulation of MYB genes during heat stress. Therefore, in-depth studies are required to elucidate the underlying biological mechanisms. Calmodulin has a significant effect on the responses of plants towards heat stress [57]. In our study, we successfully identified a gene that is regulated by calcium and is a transcriptional co-activator that responds to calcium. However, the precise contribution of this gene to the heat-stress response of *P. ternata* seedlings via miRNA regulation remains to be investigated. Integrated analysis revealed the presence of several uncharacterized proteins. This observation could be attributed to the absence of comprehensive genetic data for *P. ternata*. However, this also implies the potential existence of additional stress-related genes among the target genes. Furthermore, we constructed a molecular regulatory network of these miRNAs to depict their intricate interactions with genes, thereby offering insights into the regulatory mechanisms underpinning *P. ternata* seedlings’ response to heat stress. Nevertheless, further experimental validation is necessary to fully elucidate miRNA-mediated regulatory pathways.

## Conclusion

In summary, we studied small RNAs and their target genes in *P. ternata* using transcriptome and miRNAome profiles when exposed to heat stress. Although the complex miRNA-mediated regulatory networks require further study, our findings provide valuable information for the characterization of genes and miRNAs that respond to heat stress in *P. ternata*. Additionally, these results can be useful for other plant species, as they provide insights into the molecular mechanisms that govern plant responses to abiotic stress.

### Electronic supplementary material

Below is the link to the electronic supplementary material.

Supplementary Material 1

Supplementary Material 2

Supplementary Material 3

Supplementary Material 4

Supplementary Material 5

Supplementary Material 6

Supplementary Material 7

Supplementary Material 8

Supplementary Material 9

Supplementary Material 10

## Data Availability

The datasets presented in this study can be found in online repositories. The names of the repository/repositories and accession number(s) can be found below: NCBI GEO (https://www.ncbi.nlm.nih.gov/geo/) and GSE243965.
