# Supplementary material for: Integrated analysis of transcriptome and miRNAome reveals the heat stress response of Pinellia ternata seedlings
Source: BMC Genomics. 2024 Apr 23;25:398. doi: 10.1186/s12864-024-10318-x (PMC11040748; doi:10.1186/s12864-024-10318-x)
Supplement: Supplementary file 10 — Supplementary Material 10 [file 12864_2024_10318_MOESM10_ESM.docx]

Supplemental Figures

**Integrated analysis of** **transcriptome and** **miRNAome reveals the heat stress response of *Pinellia ternata* seedlings**

Chen Bo^1,2†^, Mengmeng Liu^1†^, Qian You^1^, Xiao Liu^1^, Yanfang Zhu^1,2^, Yongbo Duan^1,2^, Dexin Wang^3*^, Tao Xue^1,2*^ and Jianping Xue^1,2*^

^1^Anhui Provincial Engineering Laboratory for Efficient Utilization of Featured Resource Plants, College of Life Sciences, Huaibei Normal University, Huaibei 235000, China

^2^Huaibei Key Laboratory of Efficient Cultivation and Utilization of Resource Plants, College of Life Sciences, Huaibei Normal University, Huaibei 235000, China

^3^ College of Agriculture and Bioengineering, Heze University, Heze 274000, China

^†^Chen Bo and Mengmeng Liu are contributed equally to this work.

* Correspondence:

Dexin Wang

e-mail: wangdexin1996@163.com

Tao Xue
e-mail: xuetao_26@163.com

Jianping Xue
e-mail: xuejp@163.com


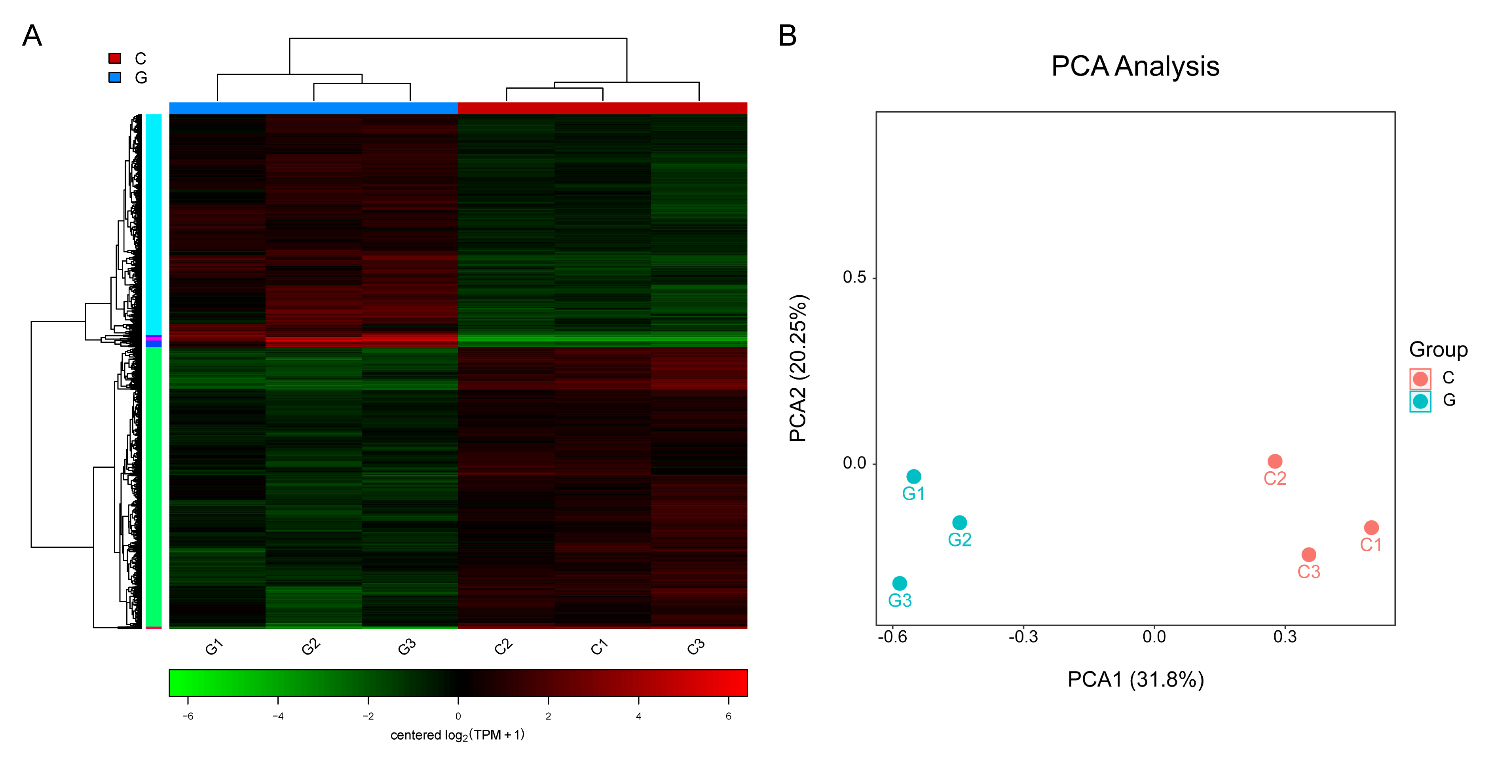


**Supplemental Fig. S1** Transcriptome sequencing analysis in *P. ternata* under heat stress. **(A)** Cluster analysis. The color indicates the relative levels of genes from low (green) to high (red). **(B)** PCA analysis plot.


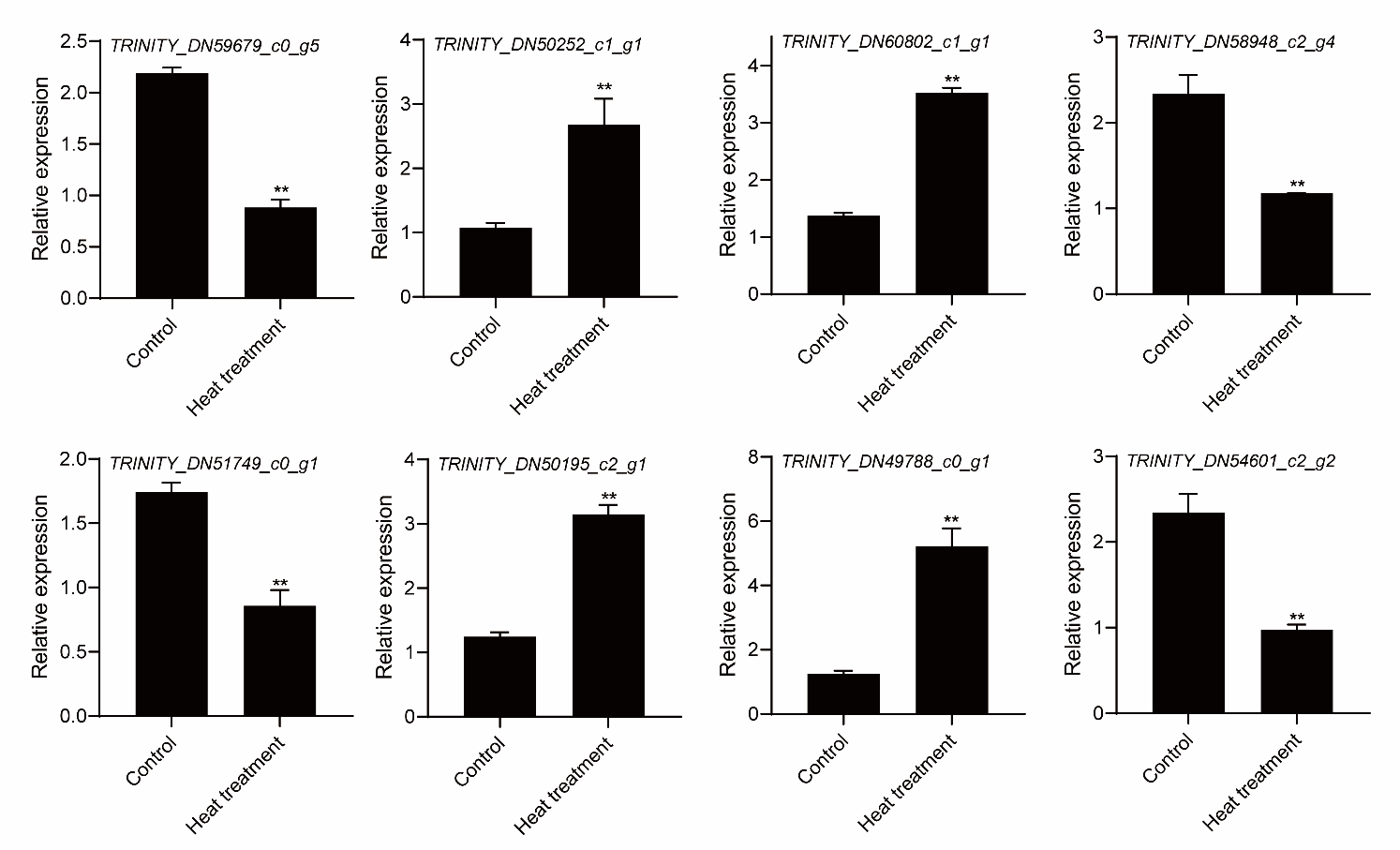


**Supplemental Fig. S2** Quantitative real-time PCR validations of DEGs characterized by transcriptome sequencing. The data is presented as mean ± SD (n = 3). The asterisks indicate significant differences based on the results of the one-way _ANOVA_ test (**P* < 0.05; ***P* < 0.01).

**
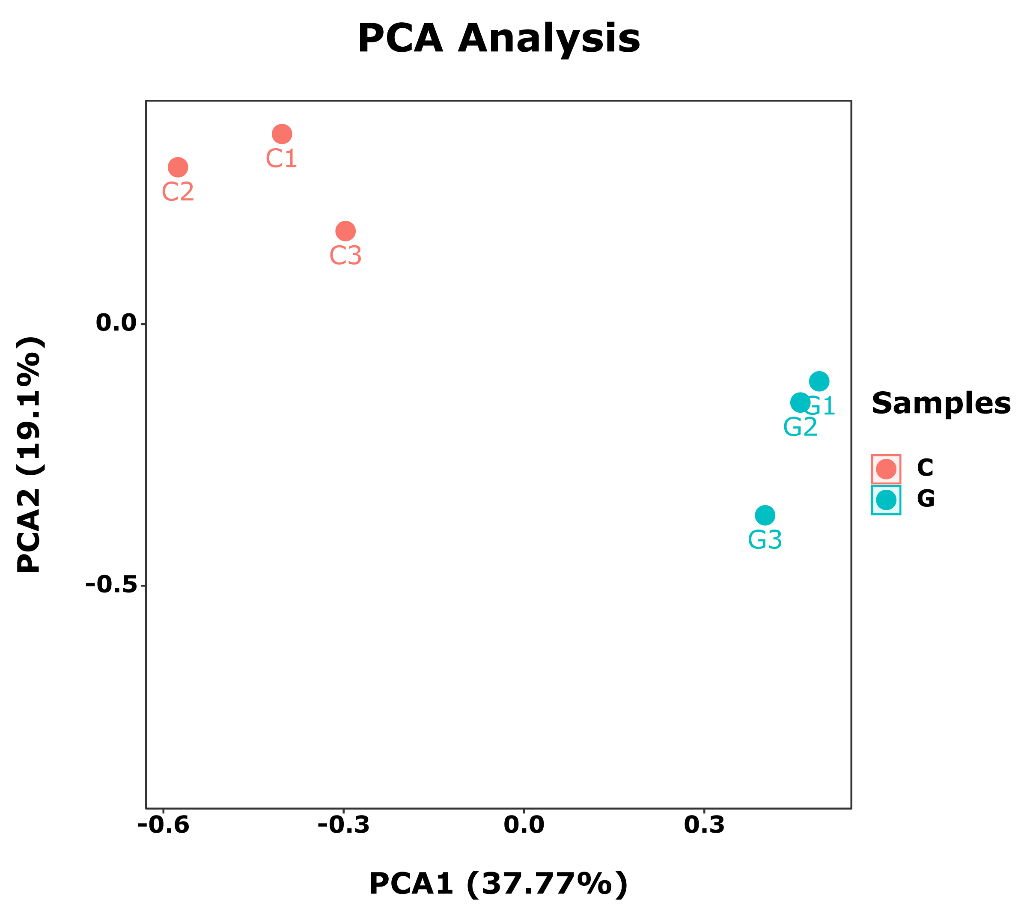
**

**Supplemental Fig. S3** PCA analysis of miRNA sequencing in *P. ternata* under heat stress.
